# Notch signaling activation is critical to the development of neuropathic pain

**DOI:** 10.1186/s12871-015-0021-0

**Published:** 2015-03-28

**Authors:** Keliang Xie, Feng Qiao, Yanyan Sun, Guolin Wang, Lichao Hou

**Affiliations:** 1 Department of Anesthesiology, Tianjin Institute of Anesthesiology, General Hospital of Tianjin Medical University, Tianjin, 300052 China; 2 Department of Orthopedics of Integrated Traditional Chinese and Western Medicine, Hong Hui Hospital, Xi’an Jiaotong University College of Medicine, Xi’an, 710054 China; 3 Department of Anesthesiology, Xijing Hospital, Fourth Military Medical University, Xi’an, 710032 Shaanxi Province China

**Keywords:** Neuropathic pain, Notch signaling pathway, Mechanical allodynia

## Abstract

**Background:**

Nerve injury-induced neuropathic pain is a major health problem worldwide. Notch signaling is a highly conserved pathway in evolution, which has an important role in synaptic plasticity and inflammation in central nervous system. The present study was designed to investigate the potential role of notch signaling in the development of neuropathic pain.

**Methods:**

The neuropathic pain was induced by spared nerve injury (SNI) in rats. The activation of notch signaling in the lumbar spinal dorsal horn was measured. DAPT, an inhibitor of notch signaling, was intrathecally (i.t.) administered before SNI or after appearance of pain sensitivity. Moreover, Jagged-1 (JAG-1) peptide, a ligand of notch signaling, was i.t. administered to normal rats. The mechanical allodynia was assessed by von Frey test.

**Results:**

Here, we found that DAPT administered 0.5 h before SNI operation could significantly prevent the decrease of mechanical paw withdrawal threshold (PWT) for more than 4 weeks (*P* < 0.05 vs. SNI group). DAPT administered after appearance of pain sensitivity could also significantly reverse the decrease of mechanical PWT in a dose-dependent manner (*P* < 0.05). In addition, administration of Jagged-1 (JAG-1) peptide significantly decreased the mechanical PWT of normal rats in a dose-dependent manner (*P* < 0.05).

**Conclusions:**

Therefore, notch signaling activation might contribute to the development of neuropathic pain. This study might provide a new therapeutic target for neuropathic pain.

## Background

Neuropathic pain is a chronic pain resulting from dysfunction or damage of the nerve fibers of peripheral or central nervous system (CNS) [1]. The mechanism is complicated and unclear, which is associated with hyperexcitability in the affected dorsal root ganglion (DRG) neurons, atrophic changes and a switch in neurotransmitter phenotype in the central afferent terminal, aberrant myelination, alterations in synaptic plasticity and excitatory and inhibitory mechanisms in spinal cord, and loss of inhibitory interneurons and modifications of brain input to spinal cord [1-7]. Although there is significant improvement in treatment, neuropathic pain frequently remains unresponsive to all treatment modalities [8]. Notch signaling is a highly conserved pathway in evolution, which often regulates cell-fate decisions in developing nervous system and has an important role in synaptic plasticity in adult CNS [9-12]. Numerous studies have demonstrated that notch signaling is crucial for many biological processes such as development, immunology, inflammation, tumor formation and memory [11,13-17]. Recent studies show that notch signaling activation might contribute to neuronal death, inhibition of neurite growth, more dendritic branching, generation and activation of microglial cells and astrocytes, differentiation of oligodendrocyte progenitors and demyelination in both peripheral nervous system and CNS [9,18-24]. Moreover, notch signaling controls the choice between excitatory and inhibitory cell fates in developing spinal cord, and its activation can promote the generation of excitatory neurons from the sensory interneuron progenitors [25]. In addition, the expression and activity of notch signaling are significantly increased after nerve injury [26]. Ligands such as Delta and Jagged bind to Notch receptors, resulting in proteolytic cleavage of Notch into two sections: an extracellular domain and a transmembrane domain. The latter cleavage is completed by the γ-secretase enzyme and ADAM, which results in the release of a Notch intracellular domain (NICD).

Therefore, we hypothesized that notch signaling activation might contribute to the development of neuropathic pain. In the present study, we firstly investigated the activation of notch signaling in the lumbar spinal dorsal horn. Then, the effects of a notch signaling inhibitor DAPT administered at different times on the mechanical allodynia were measured in a rat model of spared nerve injury (SNI)-induced neuropathic pain. Last, we studied the effects of a notch signaling activator Jagged-1 (JAG-1) peptide on the mechanical allodynia in normal rats. This study may provide a new therapeutic target for neuropathic pain.

## Methods

### Animals

All experiments were performed on adult male Sprague–Dawley rats weighing 200–250 g (Laboratory Animal Center of the Tianjin Medical University, Xi’an, China). The animals were housed in plastic boxes at 22–26°C with food and water available *ad libitum*. A 12:12 h light/dark cycle with lights on at 8:00 was maintained and testing was done between 9:00 and 18:00. Prior to experimental manipulation, animals were allowed to acclimate to the housing facilities and were handled daily at least for 3 d. All experimental protocols and animal handling procedures were approved by the Tianjin Medical University Animal Care and Use Committee and were in accordance with the guidelines for the ethical treatment of animals established by the International Association for the Study of Pain. All efforts were made to minimize animal suffering and to reduce the number of animals used.

### Intrathecal catheterization and drug delivery

A permanent intrathecal (i.t.) catheter (PE-10 polyethylene tube, BD Biosciences, Franklin Lakes, NJ) was inserted through the gap between T_3_ ~ _4_ vertebrae and extended slowly to the subarachniod space of lumbar enlargement (L_4_ and L_5_ segments) under pentobarbital sodium anesthesia [40 mg/kg, intraperitoneal (i.p.)] [27]. The catheter was filled with sterile saline (approximately 4 μL), and the outer end was plugged. All animals appeared to be free of infection through gross inspection. The cannulated animals were allowed to recover for 4 days. Motoric integrity was assessed in all animals using the righting reflex and inclined plane test. Animals showing any neurological deficits were excluded from the following experiment.

Drugs were injected over a period of 1 min via the catheter at a volume of 10 μL, followed by 5 μL sterile saline for flushing. DAPT (Sigma-Aldrich, St. Louis, MO, USA) and JAG-1 peptide (CDDYYYGFGCNKFCRPR) (AnaSpec, San Jose, CA, USA), as a potent inhibitor and activator of notch signaling, respectively, were both freshly dissolved in dimethyl sulfoxide (DMSO) at a concentration of DAPT (25, 50 or 100 μM) and at a concentration of JAG‑1 peptide (1, 10 or 100 μM) [19]. The DMSO or scrambled peptide (SC)-JAG-1 (RCGPDCFDNYGRYKYCF) (AnaSpec, San Jose, CA, USA) was used as a control. The location of the distal end of this catheter was verified at the end of each experiment by injection of pontamine sky blue via the catheter.

### Spared nerve injury (SNI) model

The neuropathic pain was induced by left SNI model as previously described [28]. Briefly, under pentobarbital sodium anesthesia, an incision was made on the lateral thigh and the underlying muscle was separated to expose sciatic nerve. The three terminal branches of sciatic nerve (tibial, common peroneal and sural nerves) were carefully separated. After separation, the tibial and common peroneal nerves were tightly ligated with 5.0 silk, and then 2–3 mm of the nerves distal to the ligation was removed. The muscle and skin incisions were then closed separately. Sham operation was performed identically without the passage of ligatures or transection of the nerves.

### Assessment of notch signaling activity

The activation of notch signaling was measured by western-blot of NICD. The lumbar spinal dorsal horn tissues were harvested, sonicated/homogenized and centrifuged. The protein was extracted from the above preparations, and loaded and separated on a 10% (w/v) SDS-polyacrylamide gel. After transfer, blots were incubated overnight at 4°C with separately with NICD (1:1000; Abcam, London, UK) and beta-actin (1:10000; Chemicon, Temecula, CA, USA) primary antibodies. The membrane was washed and primary antibodies were detected with secondary antibody conjugated to horseradish peroxidase for 1 h at room temperature. Blots were visualized using enhanced chemiluminescence (ECL; Roche, Basel, Switzerland).

### Assessment of mechanical allodynia

The mechanical allodynia was assessed by von Frey test as previous described [29]. Mechanical allodynia of rats was determined by paw withdrawal threshold (PWT) in response to mechanical stimuli produced by using a calibrated series of von Frey filaments (Stoelting, Chicago, IL, USA). The animals were adapted to the testing situation for at least 30 min before stimulation was initiated. During the test, the rats were placed on a metal mesh floor covered with the same plastic box and von Frey filaments were applied from the underneath of metal mesh floor to the lateral plantar surface of the paw (the area innervated by sural nerve). Each filament was presented perpendicularly against the paw, with sufficient force to cause slight bending, and held 2–3 s. The filament was applied only when the rat was stationary and standing on all four paws. A withdrawal response was considered valid only if the hindpaw was completely removed from the customized platform. Lifting of the paw due to normal locomotor behavior was ignored. The monofilaments were applied with increasing force until the rat withdrew the paw. Each hair was applied 10 times at 5 s intervals. The bending force value of the von Frey filament that caused a 50% occurrence of paw withdrawal reflex by 10 times stimuli was expressed as the PWT. After the threshold was determined for the left hindpaw, the same testing procedure was repeated on the other hindpaw at 5 min interval. Second and third testing trials were run for both hindpaws, respectively. If the withdrawal threshold in the second or third trial did not match the withdrawal threshold of the previous testing trial in a given hindpaw, the next large hair in the series was tested. This was done until the withdrawal threshold in the three successive trials matched. To avoid inter-experimenter differences and subjective bias, all the behavioral observations were performed by one who was blind to the treatment.

### Statistical analysis

Data are expressed as mean ± standard deviation (SD). Statistical comparisons between groups were done using univariate ANOVA or ANOVA for repeated measurements. The statistical analysis was performed with *SPSS* 16.0 software (SPSS Inc, Chicago, IL). A *P* value of less than 0.05 was considered statistically significant for all tests.

## Results

### Notch signaling is activated after SNI operation

**Figure 1:**
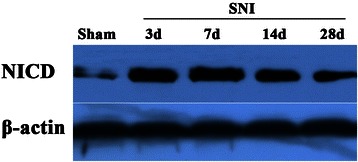
**Changes in expression of NICD in the dorsal horn of lumbar spinal cord in control and SNI model rats.** Immunoblotting showed the expression of NICD in the dorsal horn of lumbar spinal cord. NICD increased from 3 days to 28 days after nerve injury (SNI model). NICD: notch intracellular domain; SNI: spared nerve injury.

In this study, we firstly investigated the notch signaling activity at different time points after SNI operation. This present study showed that NICD expression in the dorsal horn of lumbar spinal cord was markedly increased until 28 days following peripheral nerve injury (Figure 1). This result suggests that notch signaling is activated during the development of neuropathic pain.

### Administration of DAPT before SNI operation significantly prevents the development of mechanical allodynia in neuropathic pain

**Figure 2:**
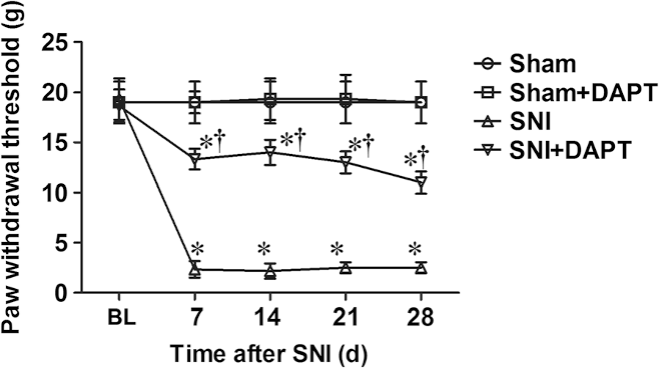
**DAPT (an inhibitor of notch signaling pathway) administered before appearance of pain sensitivity significantly prevents the development of mechanical allodynia in SNI-induced neuropathic pain.** A single administration of DAPT (50 μM, 10 μL, i.t.) was 0.5 h before SNI operation. The mechanical mechanical paw withdrawal threshold (PWT) was measured at 24 h before (baseline) as well as 7 d, 14 d, 21 d and 28 d after SNI or Sham operation. The data represent mean ± SD (n = 6 per group). ^*^P < 0.05 versus Sham group; ^†^*P* < 0.05 versus SNI group. SNI: spared nerve injury; BL: baseline; g: gram; d: day.

In the present study, we then investigated the effects of notch signaling pathway inhibitor DAPT pretreatment on mechanical allodynia in a rat model of SNI-induced neuropathic pain. In this experiment, the mechanical allodynia in all animals was measured at 24 h before (baseline) as well as 7 d, 14 d, 21d and 28 d after SNI or sham operation. The SNI-challenged animals developed a marked hypersensitivity to innocuous mechanical stimulation of the lateral surface of the hindpaw (sural nerve skin area) (Figure 2). The hindpaw contralateral to the operation was tested over the whole period and did not demonstrate any statistically significant change in mechanical PWT from pre-operation baseline (*data not shown*). The mechanical PWT from 7 d to 28 d after SNI operation decreased significantly (Figure 2, *P* < 0.05 vs. Sham group, n = 6 per group). A single administration of DAPT (50 μM, 10 μL, i.t.) 0.5 h before SNI operation significantly improved the mechanical PWT for more than 28 d after the operation (Figure 2, *P* < 0.05 vs. SNI group, n = 6 per group). In addition, we found that administration of DAPT had no effect on the mechanical PWT of normal rats (*data not shown*). This result suggests that early inhibition of notch signaling can prevent the development of mechanical allodynia in neuropathic pain.

### Administration of DAPT after appearance of pain sensitivity significantly reverses the mechanical allodynia in neuropathic pain

**Figure 3:**
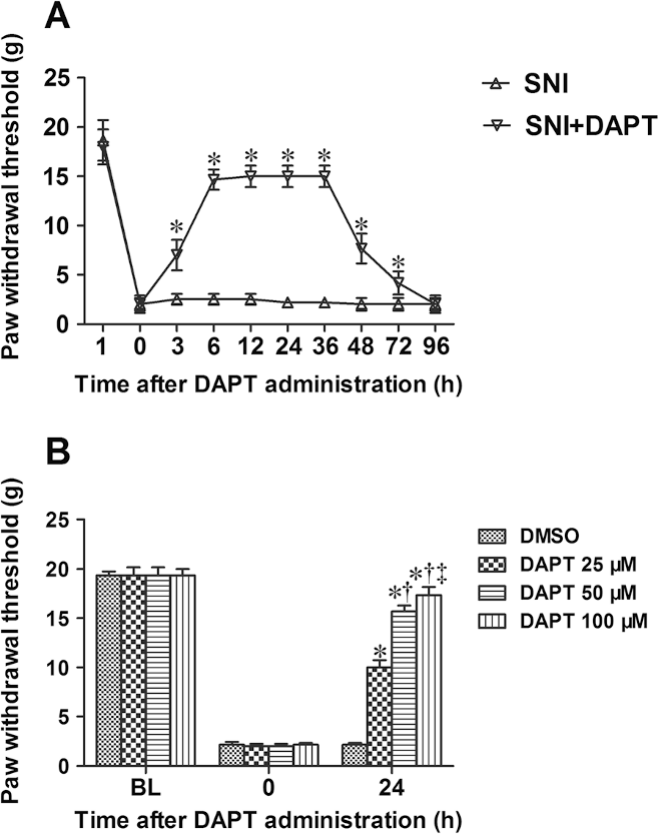
**Administration of DAPT after appearance of pain sensitivity significantly reverses the mechanical allodynia in SNI-induced neuropathic pain. (A)** DAPT (50 μM, 10 μL) or DMSO was once administrated after appearance of mechanical allodynia induced by SNI. The mechanical PWT was measured before SNI operation (baseline), before (0 h) as well as 3 h, 6 h, 12 h, 24 h, 36 h, 48 h, 72 h and 96 h after DAPT or DMSO administration. The data represent mean ± SD (n = 6 per group). *P < 0.05 versus SNI group. **(B)** Different doses of DAPT (25, 50 and 100 μM) or DMSO were administered after the appearance of mechanical allodynia. Mechanical PWT was measured prior to SNI surgery (BL), prior to administration (0 h) and 24 h following DAPT or DMSO administration. The data represent mean ± SD (n = 6 per group). *P < 0.05 versus DMSO group; ^†^*P* < 0.05 versus DAPT 25 μM group; ^‡^*P* < 0.05 versus DAPT 100 μM group. SNI: spared nerve injury; BL: baseline; g: gram; d: day, h: hour.

We then investigated the effects of notch signaling pathway inhibitor DAPT posttreatment on mechanical allodynia in SNI-induced neuropathic pain. In the preliminary experiment, we found that the animals developed significant mechanical allodynia 3 d after SNI operation (*data not shown*). A single administration of DAPT (50 μM, 10 μL, i.t.) after appearance of pain sensitivity (80 h after SNI operation) significantly increased the mechanical PWT from 3 h to 72 h after DAPT administration (Figure 3A, *P* < 0.05 vs. SNI group, n = 6 per group). Moreover, different concentrations of DAPT (25, 50 and 100 μM) were administered after the appearance of pain sensitivity, and the mechanical PWT was evaluated 24 h after DAPT administration. As shown in Figure 3B, administration of DAPT dose‑dependently increased the mechanical PWT of neuropathic pain rats (P < 0.05). These results suggest that late inhibition of notch signaling pathway can also reverse the mechanical allodynia of neuropathic pain in a dose-dependent manner.

### Administration of Jagged-1 peptide, a ligand of notch signaling pathway, can induce neuropathic pain-like behavior in normal rats

**Figure 4:**
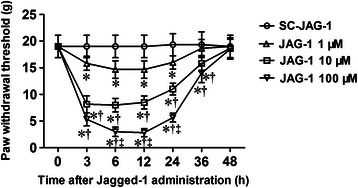
**Administration of Jagged-1 peptide, a ligand of notch signaling pathway, can induce neuropathic pain-like behavior in normal rats.** The Jagged-1 (JAG-1) peptide (1, 10 and 100 μM) or control scrambled peptide (SC)-JAG-1 were i.t. administered to normal rats. The mechanical PWT was measured before (baseline) as well as 3 h, 6 h, 12 h, 24 h, 36 h and 48 h after JAG-1 or SC-JAG-1 administration. The data represent mean ± SD (n = 6 per group). *P < 0.05 versus SC-JAG-1 group, ^†^*P* < 0.05 versus JAG‑1 1 μM group; ^‡^*P* < 0.05 versus JAG‑1 10 μM group. BL: baseline; g: gram; h: hour.

To further investigate the role of notch signaling in neuropathic pain, the JAG-1 peptide, a ligand of notch signaling pathway, were i.t. administered of 1 μM, 10 μM, 100 μM to normal animals, and the mechanical PWT was measured 3 h, 6 h, 12 h, 24 h, 36 h, and 48 h after JAG-1 administration. The SC-JAG-1 peptide was administered as a negative control. As shown in Figure 4, a single administration of JAG-1 peptide dose-dependently decreased the mechanical PWT of normal animals from 3 h to 36 h when compared with SC-JAG-1 peptide group (*P* < 0.05, n = 6 per group). The decreased PWT after the injection of JAG-1 peptide was restored 48 h later. The results further suggest that activation of notch signaling contributes to the development of neuropathic pain.

## Discussion

In the present study, we firstly found that notch signaling in dorsal horn of lumbar spinal cord was activated during the development of neuropathic pain. Early inhibition of notch signaling can prevent the development of SNI-induced neuropathic pain. Late inhibition of notch signaling can reverse the mechanical allodynia of neuropathic pain. Furthermore, activation of notch signaling can induce the mechanical allodynia of normal animals. Therefore, notch signaling activation might be critical to the development of neuropathic pain.

Injury in nervous system may result in chronic neuropathic pain characterized by increased sensitivity to painful stimuli (hyperalgesia), the perception of innocuous stimuli as painful (allodynia) and spontaneous pain [2,8]. It is well known that a characteristic symptom of neuropathic pain is mechanical allodynia [8]. The SNI model has proved to be robust, with substantial and prolonged changes in mechanical sensitivity that closely mimic many features of clinical neuropathic pain [28,29]. The model may provide a useful tool for identifying the underlying mechanisms involved in the development of neuropathic pain and as an additional screen for the efficacy of new treatments. In this study, we found that all the animals developed significant mechanical allodynia in SNI-induced neuropathic pain, which is consistent with other studies [28,29]. Many potential mechanisms have been studied which may contribute to the pathogenesis of neuropathic pain. It has been reported that multiple mechanisms at multiple sites may operate either alone or together or at different time courses to produce the complex clinical characteristics [8]. These include changes in terminal and peripheral sensitization, phenotypic switches and excitability of injured axons, hyperexcitability in the affected DRG neurons, aberrant myelination (splitting, detachment and loss of myelin), synaptic plasticity in spinal cord, loss of inhibitory interneurons and modifications of brain stem input to spinal cord, and so on [1-7]. Alterations in the balance of number and/or transmission properties of these excitatory and inhibitory interneurons are thought to be major contributing factors for chronic sensory neuropathies such as hyperalgesia and allodynia [30,31]. In addition, sensory information from the periphery is integrated and transduced by excitatory and inhibitory interneurons in the spinal dorsal horn. However, the key mechanisms that control the induction and maintenance of neuropathic pain remain unclear.

Notch is a cell-surface receptor that regulates cell-fate decisions in developing nervous system and has important roles in synaptic plasticity in adult CNS [9-11]. Binding of ligands such as Delta and Jagged results in proteolytic cleavages of notch: first in an extracellular domain and then in a transmembrane domain [13,14]. The latter cleavage is accomplished by the γ-secretase enzyme complex, resulting in the release of a notch intracellular domain (NICD) that translocates into the nucleus, where it regulates transcription [13,14]. All of the different components necessary for notch signaling, such as ligands, receptors, and enzymes involved in notch receptor cleavage, are expressed in the adult CNS as well as increase significantly after nerve injury and participate in its reparation [11,26]. Notch signaling is a highly conserved pathway in evolution, which is crucial for many biological processes such as development, immunology, inflammation, vasculogenesis, tumor formation, and learning and memory [11,13-17]. Recent findings suggest notch signaling activation can contribute to generation and activation of microglial cells and astrocytes, inhibition of neurite growth, more dendritic branching, differentiation of oligodendrocyte progenitors and demyelination in both peripheral nervous system and CNS [9,18-24]. Moreover, notch signaling pathway controls the choice between excitatory and inhibitory cell fates in the developing spinal cord, and its activation can promote the generation of excitatory neurons from the sensory interneuron progenitors [25]. To investigate the roles of notch signaling in neuropathic pain, a gamma-secretase enzyme (a key enzyme of notch signaling pathway) inhibitor DAPT was i.t. administered before or after appearance of pain sensitivity in the rat model of SNI-induced neuropathic pain. The results showed that early or late inhibition of notch signaling could prevent or reverse the mechanical allodynia of rats with neuropathic pain in a dose-dependent manner. In addition, administration of JAG-1 peptide (a ligand of notch signaling pathway) could induce the mechanical allodynia in normal rats in a dose-dependent manner. These results suggest that notch signaling activation contributes to the development of neuropathic pain.

The notch family includes 4 receptors with different functions, as indicated by the phenotypes of various notch transgenic mouse lines. These 4 receptors are expressed unequally in various cell types. Notch receptors and ligands have variable expression in various cell types. The notch signaling in the sciatic nerve, spinal dorsal horn and DRG neurons is markedly increased following peripheral nerve injury. Therefore, the notch signaling in neurons should have an important role in the development of neuropathic pain. DAPT is a non-specific inhibitor of notch receptors. Therefore the study does not specify which notch receptor(s) are involved in allodynia. Moreover, Jagged 1 is also a non-specific ligand of Notch receptors. Moreover, Jagged 1 may have different affinities for different notch receptors, depending on cellular context and post-translational modifications. Also, the extracellular Jag1 peptide will activate notch signaling indiscriminately in neurons (both inhibitory and excitatory) and glia. However, one must keep in mind the widespread and varied expression of notch receptors and ligands. What will be the effect of a systemic inhibition of notch signaling on other events where notch plays a key role in stem cell renewal? For example, hippocampus neurogenesis, that plays a key role in memory, will be also inhibited by notch inhibition.

### Limitations of this study

The notch family includes 4 receptors with different functions. DAPT is a non-specific inhibitor of notch receptors. Therefore the study does not specify which notch receptor(s) are involved in allodynia. Moreover, Jagged 1 is also a non-specific ligand of notch receptors. Moreover, Jagged 1 may have different affinities for different notch receptors. Also, the extracellular Jagged 1 peptide will activate notch signaling indiscriminately in neurons (both inhibitory and excitatory) and glia. Moreover, the roles of notch signaling pathway in thermal or cold allodynia of neuropathic pain are still further studied. In addition, its roles in inflammatory pain are unclear. The underlying mechanisms are also the future research direction.

## Conclusion

In conclusion, notch signaling activation might contribute to the development of neuropathic pain. This study might provide a new therapeutic target for neuropathic pain.

### Key message

1. Notch signaling in spinal dorsal horn is activated in neuropathic pain.
2. Early inhibition of notch signaling pathway prevents the mechanical allodynia in neuropathic pain.
3. Late inhibition of notch signaling pathway reverses the mechanical allodynia in neuropathic pain.
4. Notch signaling activation induces the mechanical allodynia in normal animals.

## Abbreviations

CNS: Central nervous system
DMSO: Dimethyl sulfoxide
DRG: Dorsal root ganglion
i.t.: Intrathecal
JAG-1: Jagged-1
NICD: Notch intracellular domain
PWT: Paw withdrawal threshold
SC-JAG-1: Scrambled Jagged-1
SNI: Spared nerve injury
